# Autoimmunity and SLE: Factual and Semantic Evidence-Based Critical Analyses of Definitions, Etiology, and Pathogenesis

**DOI:** 10.3389/fimmu.2020.569234

**Published:** 2020-10-06

**Authors:** Ole Petter Rekvig

**Affiliations:** ^1^Department of Medical Biology, Faculty of Health Sciences, UiT The Arctic University of Norway, Tromsø, Norway; ^2^Fürst Medical Laboratory, Oslo, Norway

**Keywords:** systemic lupus erythematosus, anti-dsDNA antibodies, lupus nephritis, syndrome, semantics

## Abstract

One cannot discuss anti-dsDNA antibodies and lupus nephritis without discussing the nature of Systemic lupus erythematosus (SLE). SLE is insistently described as a prototype autoimmune syndrome, with anti-dsDNA antibodies as a central biomarker and a pathogenic factor. The two entities, “SLE” and “The Anti-dsDNA Antibody,” have been linked in previous and contemporary studies although serious criticism to this mutual linkage have been raised: Anti-dsDNA antibodies were first described in bacterial infections and not in SLE; later *in* SLE, viral and parasitic infections and in malignancies. An increasing number of studies on classification criteria for SLE have been published in the aftermath of the canonical 1982 American College of Rheumatology SLE classification sets of criteria. Considering these studies, it is surprising to observe a nearby complete absence of fundamental critical/theoretical discussions aimed to explain how and why the classification criteria are linked in context of etiology, pathogenicity, or biology. This study is an attempt to prioritize critical comments on the contemporary definition and classification of SLE and of anti-dsDNA antibodies in context of lupus nephritis. Epidemiology, etiology, pathogenesis, and measures of therapy efficacy are implemented as problems in the present discussion. In order to understand whether or not disparate clinical SLE phenotypes are useful to determine its basic biological processes accounting for the syndrome is problematic. A central problem is discussed on whether the clinical role of anti-dsDNA antibodies from principal reasons can be accepted as a biomarker for SLE without clarifying what we define as an anti-dsDNA antibody, and in which biologic contexts the antibodies appear. In sum, this study is an attempt to bring to the forum critical comments on the contemporary definition and classification of SLE, lupus nephritis and anti-dsDNA antibodies. Four concise hypotheses are suggested for future science at the end of this analytical study.

## Introduction

**Figure 1:**
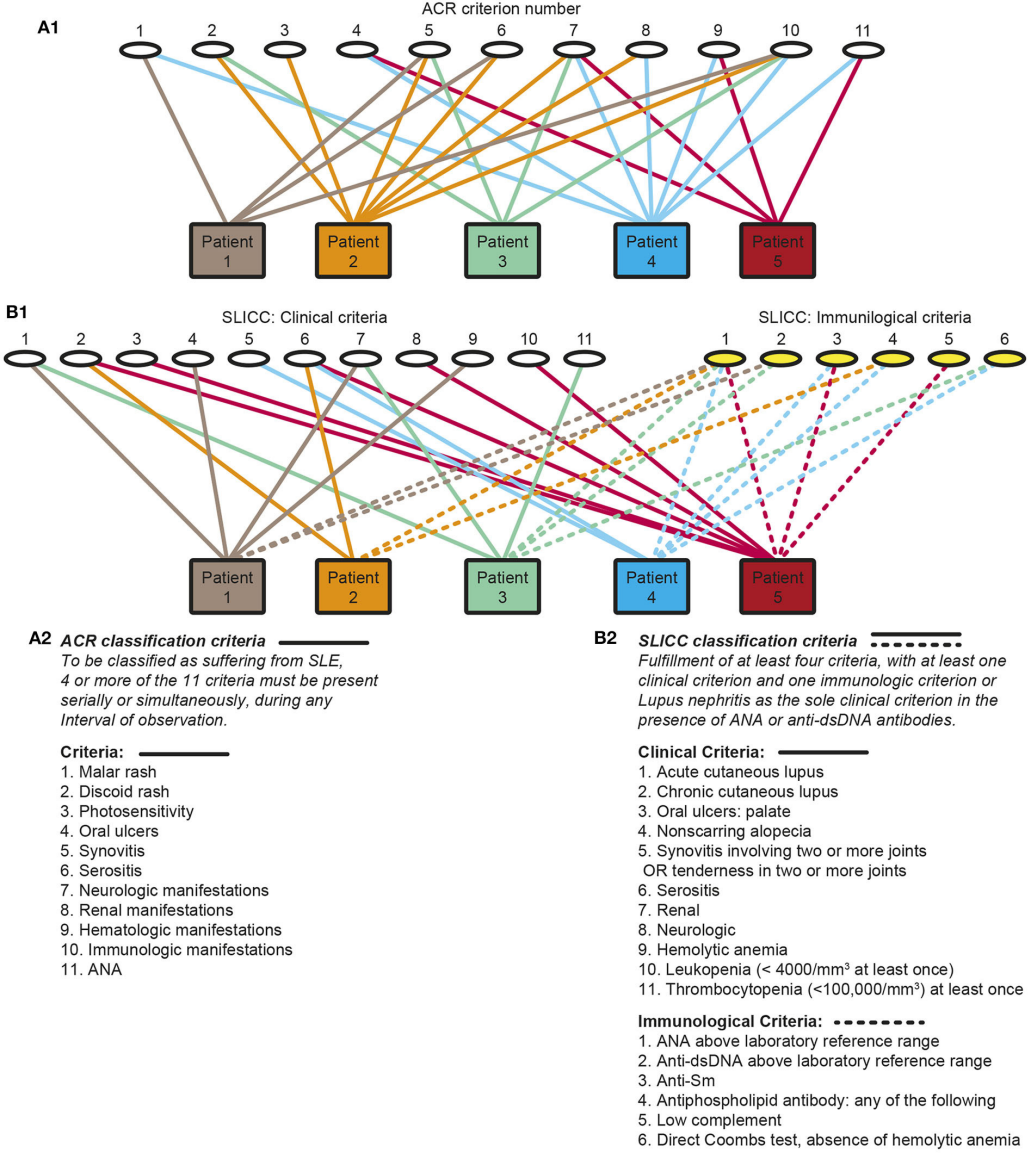
Principal problems linked to classification of systemic lupus erythematosus (SLE). Classification of SLE patients according to The American College of Rheumatology (ACR) **(A1,A2)** or by The Systemic Lupus International Collaborating Clinics Criteria (SLICC) **(B1,B2)** classification criteria are descriptively problematized. Each of the classification systems identify a substantial diversity of clinical phenotypes. The 11 ACR criteria is presented by numbers (**A1**, the classification criteria are presented as a focused table in **A2**). Five patients are demonstrated. The patients share some criteria, but diverge with respect to others, and their clinical phenotypes differ individually. Similarly, each of 11 clinical and 6 immunological SLICC criteria are presented by numbers (**B1**, the classification criteria are presented as a focused table in **B2**). These chaotic figures **(A1, B1)** demonstrate that the use of the ACR and the SLICC criteria is problematic as bases for scientific analyses covering genetics, etiology, pathogenesis, and response to experimental therapy in patient cohorts as the study objects do not represent a homogenous group of patients. The patients in these figures are fictive but they reflect problems with the ACR and SLICC criteria in real life (Part of this figure (A) is a reprint with permission of Figure 1 in Rekvig (3).

SLE, lupus nephritis and anti-dsDNA antibodies represent cores of this, in principal eclectic study. The narrative is in its nature a critical view on definition of lupus nephritis as part of the syndrome SLE, and its classification, etiology and pathogenesis. In particular, the interrelationship between numerous classification criteria has not been given priority in the literature, notably not in the original manuscripts presenting the 1982 American College of Rheumatology [ACR (1)] and the 2012 Systemic Lupus Erythematosus International Collaborating Clinics [SLICC (2)] sets of classification criteria. In the introduction to the revised SLICC SLE classification criteria it is stated “*To ensure that there is a consistent definition of SLE for the purposes of research and surveillance, classification criteria for SLE are needed*” (2). This statement indicates that the ACR or SLICC classification criteria are valid as reliable approaches to define SLE, even though they do not define SLE as a homogenous disease since the classification criteria by definition provides hundreds of clinical phenotypes [discussed in (3)]. Figure 1 principally demonstrates the clinical phenotype variability problem. One basic problem is that the SLE study objects—the patients—are included based on selected heterogeneous clusters of classification criteria as defined in the 1982 ACR (1), the 1997 revised ACR (4), the 2012 SLICC criteria (2) and recently the 2019 EULAR/ACR classification criteria for SLE (5) instead of selecting cohorts of patients with a homogenous phenotype like lupus nephritis and anti-dsDNA antibodies as selection parameters.

**Figure 2:**
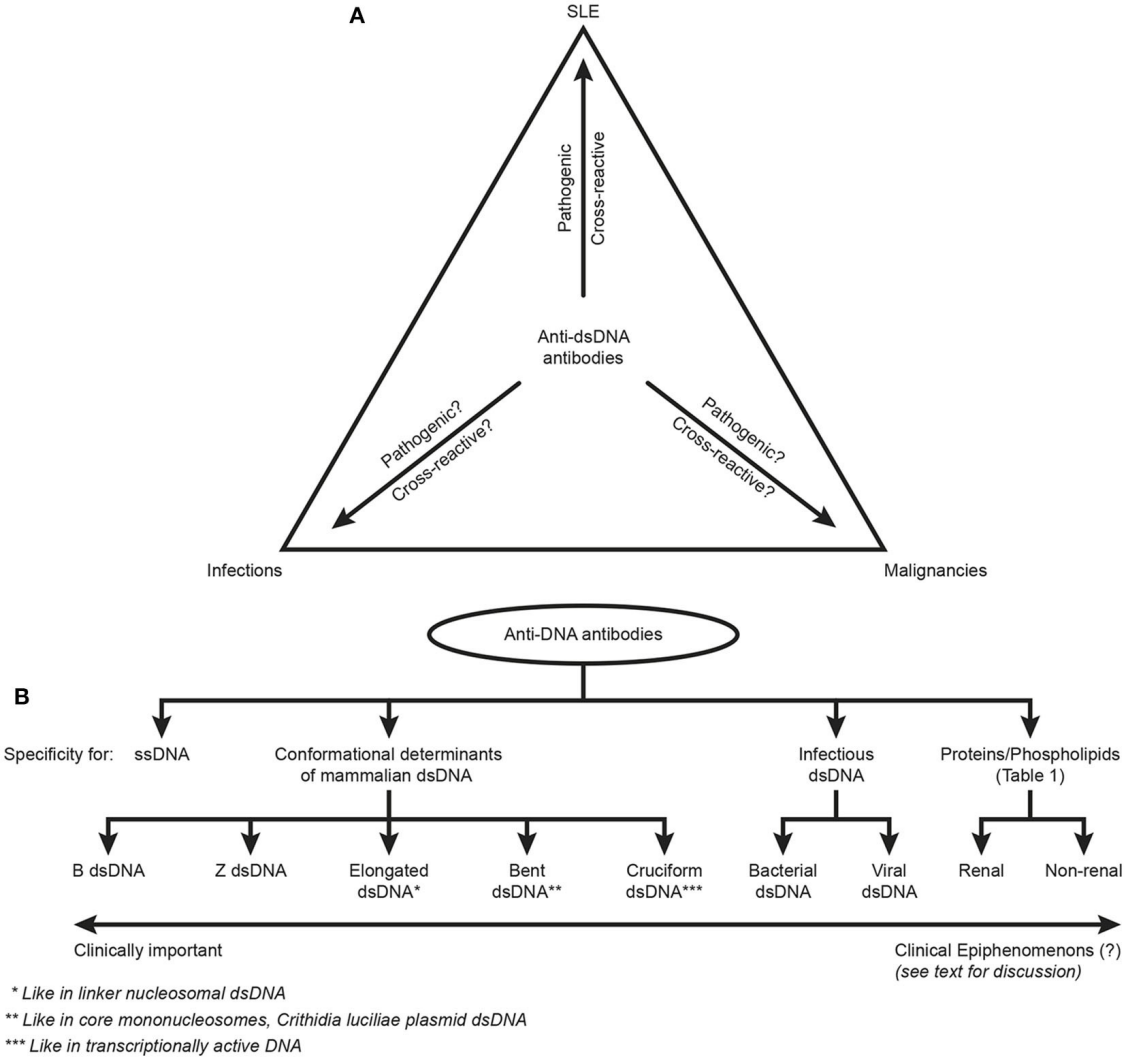
Principal problems linked to the ACR and SLICC classification criterion “The anti-dsDNA antibody” (criterion 11 in ACR) or “Anti-dsDNA” (criterion 6, Immunological criteria, SLICC). Principal simplified problems are linked to the inadequate terminology of the anti-dsDNA antibodies. One problem [demonstrated in **(A)**] illustrates that “The anti-dsDNA antibody” is not unique for SLE, but appears regularly in context of infections and malignancies (see text for details). Little is examined whether anti-dsDNA antibodies are pathogenic and cross-reactive in the latter two categories of conditions (question marks in **A**) as they are in SLE. This triangular nature of anti-dsDNA antibodies has not been considered in the classification criteria, and poorly in the relevant literature on SLE. The second dominant problem considered for the “Anti-dsDNA antibody” is that the antibodies are presented as if “it” is monospecific for dsDNA. This has over decades crystallized the conception that different assay systems detect antibodies possessing different *avidities* but not different *specificities*! This conflict is principally demonstrated in **(B)**. The “ssDNA/dsDNA” is categorized in 4 main categories with 5 subcategories for mammalian dsDNA, 2 for infectious dsDNA and 2 for cross-reaction of anti-dsDNA for renal and non-renal proteins/phospholipids (see Table 1 for details on the latter category). Antibodies for all these dsDNA structures have been identified by conventional assay systems, like ELISA in physiological salt (elongated/bent B dsDNA), in high salt (Z dsDNA), cruziform dsDNA, bacterial and viral dsDNA in addition to heterogeneous binding to proteins and phospholipids. The idiom that anti-dsDNA antibodies bind dsDNA in a singular form as in the ACR or SLICC classification systems must be challenged by the multifaceted recognition pattern of anti-dsDNA antibodies as informed in **(B)**. Thus, data in this figure require that assay systems for anti-dsDNA antibodies relates to categorized structural dsDNA specificities. Lack of implementation of the structural and molecular recognition pattern recognized by individual anti-dsDNA antibodies undermine the potential clinical impact of anti-dsDNA antibody sub-specificities.

This critical argumentation is not equally relevant to studies on elements of systemic autoimmunity, like autoimmunity to dsDNA in SLE [see e.g., (3, 6–16)]. Such studies are focused on distinct autoimmune processes that are unlinked from a solitary SLE context, as is indicated by the triangular^1^ link of anti-dsDNA antibodies to SLE, infections and malignancies (Figure 2A). Autoimmunity to chromatin structures is, however, *relevant* for SLE (11, 13, 14, 35–38), and for pathogenesis of organ manifestations like lupus nephritis, dermatitis and cerebral affections, as discussed below.

Paradoxically, we are not able to explain *why* the classification criteria by any combinations *define* SLE. The criteria are neither etiologically nor pathogenetically linked to each other, a problem that has not been seriously discussed [see published discussions in (1, 2, 5)]. In the context, lupus nephritis may robustly stand on own feet as a unique and identifiable disease, as unintentionally (?) indicated in the SLICC criteria, as this set of criteria says that a person may have SLE if positive for anti-dsDNA antibodies and demonstrating proteinuria. Thus, we are not able to provide a concise definition of SLE and lupus nephritis, but we identify SLE when we encounter patients. This is based on inconsistent rather than coherent classification criteria, intuition, and on experience.

## Systemic Lupus Erythematosus—the Syndrome

SLE is an enigmatic disease, in which little of its pathogenesis and less of its etiology is understood. In the history of SLE, it is not possible to recognize penetrating studies that focus on an autoimmune origin (in sense of etiology) of SLE, but autoimmunity is recognized as a *disease-modifying* factor (in sense of pathogenesis) that promote disease progression (3, 39–41). Rather, genetics in humans (42–44) and in mice (45, 46), infections (13, 15, 47–53), or cancers (13, 54–57) may be relevant research foci to study molecular processes accounting for etiology. The transformation of etiology into pathogenic autoimmune processes are regarded central to understand the imaginative syndrome SLE.

### SLE: Syndrome, Etiology, and Pathogenicity—Clarifying the Terms (Lexical and Logic Semantics and Simplifications)

Three terms are used to describe SLE: Syndrome, etiology, pathogenesis. The term *syndrome* means concurrence—symptoms appearing simultaneously. Etiology comes from etymologistic: the study of causation, origination from the Greek αἰτιoλιγíα, aitiología, “giving a reason for” (αἰτíα, aitía, “cause”; and -λoγíα, -logía) (58). Etiology means the predisposition of a disease or syndrome, and therefore something that promotes pathophysiologic processes. Pathophysiology means the origination and development of a clinical disease. If etiology means the basic initiator, pathogenesis means the effector of the disease. These terms are important to consider if we aim to understand how to categorize hypotheses, basic and clinical science on SLE—and how to probe hypotheses aimed to understand the impact of classification criteria. There exists no evidence that SLE is promoted by an autoimmune etiology, because the 11 ACR or 17 SLICC criteria are definitively not connected to a common etiology. The criteria may per statements appear cumulatively in the body at different time points as specified (1, 2) and interpreted in the study of Arbuckle et al. (59). Considering the highly diverse nature of individual classification criteria, the criteria may in fact rely on different etiologies, and consequently on different pathogenic processes. The autoimmune pathogenesis involved in evolution of the syndrome SLE may therefore be set in motion not by a uniform underlying etiology, but by etiologies promoting individual classification criteria. A definition of SLE as a syndrome (3, 12, 39, 60) is therefore etymologically and theoretically unjustified. There are few discussions related to this problem, but are tangentially approached by Touma et al. (61).

Furthermore, accepting that criteria like “The anti-dsDNA antibody” may appear timely unlinked from a clinically overt pathogenic process challenges the Witebsky postulates attempting to define a disorder as autoimmune and pertained by a specific autoimmune response (62, 63) in analogy to the Koch's postulates to define a causative relationship between a defined microbe and a consequently defined disease^2^. The essence of the Witebsky postulates is that an autoantibody account for a given tissue damage, and the characteristic pathological changes can be reproduced upon transfer of the autoantibody (or suspected T cells) into experimental animals. This is discussed below.

### SLE: A Short History of Non-linear Periodic Paradigm Shifts Leading to Our Times Syndrome

The syndrome originally being described as a skin disease in antiquity has evolved into a complex disease through milestones defined as non-linear paradigm shifts (64–66). What can we learn from this still ongoing evolution of SLE, and how can we include the term SLE into a scientifically insinuated disease entity? In this context, the complexity of SLE and patients suffering from SLE has been thoughtfully and eruditely presented by David Isenberg (67).

The transformation of SLE from a serious monosymptomatic skin disease into a syndrome has in Ludvik Fleck's (68) and Thomas Kuhn's (69) sense not evolved linearly, but through radical paradigm shifts. The central milestones appeared after studies in the 1850ies and their aftermaths. Central perceptions derive from paradigms settled by Cazenave in 1850ies (70), and later by Kaposi (71), Osler (72, 73), and Jadassohn [(74), reviewed in (64, 65, 70, 75)]. Through these paradigm shifts, the definition of SLE has evolved into that of a syndrome including systemic affection of viscera (71, 73) and later also comprising immunological, biochemical and hematological parameters (1, 2, 5). This has resulted in new authoritarian descriptions of SLE as in the canonical ACR (Figure 1A) or SLICC (Figure 1B) sets of criteria. With still ongoing expansion of classification criteria we have not reached a consensus on what SLE is, or what its etiology or pathogenesis are. Thus, the paradigm shifts resulting in the modern perception of SLE has not been very helpful to understand what SLE basically is, but they provided an understanding of its complex and systemic nature. An intriguing question we can raise in this context is if SLE of today at all is the disease known in antiquity as a skin disease.

When SLE should be interpreted from principals as those observed, implemented and decoded in the classification criteria, the *positivists*^3^ would (and indeed do so!) describe the syndrome through collection of facts/criteria. The elements that exert this collection of facts have, however, not reached a logic description based on firm scientific data beyond statistical co-appearance - cumulatively or simultaneously. By this, the syndrome SLE can, as it is understood today, be classified by a hermeneutic^4^ approach to understand its nature.

### SLE: A Primary or Secondary Autoimmune Syndrome; Etiology vs. Pathogenesis

A classical statement promotes SLE as a prototype complex autoimmune syndrome (1, 2, 5, 61, 76, 77). However, this statement is standing in a certain contextual, but contrafactual paradigm hampered by one central logical problem: We do not understand an etiological origin of the classification criteria, or what the link between the current criteria are. We have till now not determined if they at all emerge from an inner biological coherence. Theoretically, they might be determined by a common etiology, or by diverse pathogenic processes that account for the apparently non-coherent classification criteria. If we aim our studies to understand the meaning of all the classification criteria for SLE, we need to distinguish the syndrom's etiology from the (secondary) pathogenicity that account for the manifold of the syndrome's classification criteria.

The latter statement has not been profoundly discussed in the literature. Only few exceptions from this offensive comment on the classification criteria have been discussed. One obvious exception is expressed in the SLICC criteria; a patient is said to have SLE if having two criteria fulfilled: nephritis (proteinuria) concurrently with anti-dsDNA antibodies (2). Here, the antibody is strategically and logically linked to renal inflammation in a causal relationship: The antibody as inducer of de facto renal inflammation in accordance with the Witebsky proposals. The other comes from a study published by Pisetsky et al. (77) where they introduce a principal system for categorization of SLE phenotypes; i.e., defining phenotypes of SLE in groups according to interrelated criteria to define subgroups of SLE. In fact, Pisetsky's suggestion resembles data from Isenberg et al. where they upon longitudinal studies of 988 SLE patients identified different clusters of phenotypes (76). The newly suggested revision of the criteria published by Aringer et al. (5) and Touma et al. (61) do not help much here, as these revised criteria cement non-interrelated affections into an enigmatic disease entity! This is recently critically analyzed and discussed by Petri et al. (78).

### SLE: A Cumulative Model for the Classification of SLE Raises Problems Linked to the Terms Etiology and Pathogenesis

Relevant in the present context is to understand what ties the evolving number of classification criteria together aimed to classify the *syndrome* SLE—a common etiological *or* a common pathogenetical mechanism? Or are they tied together as a result of a domino effect of pathogenic events: one leads to other events that are not initiated by the primary etiology? And what is the rationale behind the statements in the classification criteria that any events (processes, clinical criteria, deviating laboratory parameters) counts over the timeline of the syndrome. Classification criteria that appear disparate in time count cumulatively. According to the definition of the term syndrome—concurrence—this term does not harmonize with the statement that the criteria may appear simultaneously or at any time point in the history of a patient. If the criteria are related to each other as inducers (autoimmunity?) or responders (organ affections?) then how can the one or the other appear disparate over years? This is an accepted, although contrafactual, statement in the classification criteria which is not in agreement with the Witebsky postulates to define a disease as caused by a specific autoimmune antibody or an autoimmune T cell.

On the other hand, an autoimmune pathogenesis of SLE may be a valid term for some of the criteria (like lupus nephritis or lupus-related skin and cerebral affections) characterizing SLE. In harmony with this, data demonstrate that the kidney disease evolves and is maintained (*pathogenesis*), but not proven to be initiated (*etiology*) by autoimmune responses with anti-dsDNA antibodies as the central pathogenic factor (11, 16, 33, 79–81). However, other criteria than lupus nephritis, lupus dermatitis (82), and certain cerebral affections (83), have pathogenic origins that are beyond the impact of anti-dsDNA antibodies. It may be wise to probe the term autoimmune pathogenesis with the Witebsky postulates (62, 63) to establish a causative relationship between a specific autoimmune response and a subsequent autoimmune disease.

Another principal problem related to the use of classification criteria is based on epidemiological studies and studies on the effect of experimental therapeutic modalities. A critical question must therefore be if patients implemented in multicenter-based ACR or SLICC defined cohorts are homogenous to a degree that allow us to validate results related to basic aspects of SLE, like its etiology, pathogenesis, epidemiology and effect of experimental therapy. This somewhat pedantic discussion is important since SLE is regarded as an integrated and unified syndrome—however without parameters that justify this assumption.

## Current Approaches to Study the Nature of SLE

The contemporary ACR or SLICC criteria-related definitions of SLE and its canonical link to autoantibodies against dsDNA (10, 13, 14) can be confronted by argumentations at different theoretical levels;

*i*. Do we have clear evidence-based definitions of the syndrome and its marker antibodies;

Our contemporary insight into the syndrome SLE derives from three mainstream types of scientific approaches. One is based on identifying basic hypotheses related to separate processes accounting for individual classification criteria. The second approach is aimed to analyse why a wide diversity of clinical, biological, and biochemical parameters in SLE cohorts are implemented as diagnostic and classification measures. The third is a neglected approach; lack of studies to elucidate why the diverse classification criteria are appearing clustered in SLE. These approaches have not guided us into evidence-based definitions of SLE and its canonical marker antibodies. If we are going further into these problems, we do not need to generate more classification criteria, we need to select conservative and uniform selection criteria in order to implement homogenous patient cohorts, like those positive for proteinuria and anti-dsDNA antibodies. By this, we can analyse whether these two selection criteria define SLE and classification criteria that are pathogenetically linked to nephritis and anti-dsDNA antibodies. To select cohorts based on all combinations of classification criteria, as demonstrated in Figure 1, may yield some statistically significant combination of symptoms/parameters, but not information on pathogenesis and even less on etiology of each criterium or SLE itself.

*ii*. Do we perform sound theoretical considerations applied to etiology and to pathogenesis of the syndrome itself as opposed to its individual criteria;

Such requested studies are difficult to identify in historical or contemporary studies. One possible approach could be to identify analytically or through studies of relevant literature etiological and pathogenetic processes accounting for individual classification criteria.

*iii*. Can we implement open-minded reservations in this argumentation, or is this approach dominated by dogmatic conclusions deriving from statistical data (the positivistic approach)?

In my opinion we have to generate clear reservations when implementing newly and previously defined classification criteria. If statistically significant associations of criteria should be weighted, then biological and pathogenical studies must be performed to promote information as to why these criteria tell us something about SLE.

Therefore, prevailing limitations of contemporary cohort studies are founded on analyses of highly heterogenic groups of SLE patients [discussed in (3)]. This simple fact makes studies of SLE difficult without clearly defined and reflected hypotheses (see suggested hypotheses in the conclusion section).

## What may Emerge From These Theoretical Tribulations and Considerations?

A conclusion of these reflections and concerns is that we need to reconsider how we classify SLE. We also need to generate new testable hypotheses, and accordingly to perform studies on clinically homogenous patient cohorts, and to define biomarkers relevant for such homogenous cohorts of SLE patients. Basically, we need to determine whether revised or contemporary classification criteria for SLE are etiologically or pathogenetically logic and understandable. In sum, we must prioritize, or categorize according to Pisetsky's definition (77), the criteria to approach a more uniform and homogenous definition of the syndrome SLE. For example, a homogenous cohort could be patients demonstrating anti-dsDNA antibodies concurrently with proteinuria, taking only these two criteria into account. In that context it would be intriguing to observe which of clinical or laboratory parameters would deviate from normal values.

## “The Anti-DSDNA Antibody” - an Account to its Nature and Structural DNA Specificities

This heading indicates a problem. “The anti-DNA antibody,” as defined in ACR or SLICC classification criteria, is just that, and does not reflect anti-dsDNA antibodies specific for various dsDNA structures (see Figure 2B). This statement underscores the problems defined in the following proclamations. Anti-dsDNA antibodies occur in SLE, are a classification criterion for SLE, exist in autoimmune syndromes other than SLE (13), in bacterial (48, 53, 84), viral (49, 85–87), and parasitic infections (88), and in cancers [(89), see Figure 2A). Importantly, these sets of anti-dsDNA antibodies have multiple specificities for unique DNA structures (Figure 2B). They have a pathogenic impact in SLE (but not in infections or in cancers?), and they may even be detected in healthy individuals [see general discussions in (10, 13, 14)].

### Anti-dsDNA Antibodies: Appearing in Principally Different Clinical Conditions

The annexation of “The anti-DNA antibody” as a criterion for SLE does not communicate its pertinent clinical impact aside from simply being claimed to be involved in lupus pathogenesis [although how is still disputed (11)] or in which circumstances the antibodies are clinical epiphenomena distinctively separated from their assumedly pathogenic effects or their status as biomarker. See in this context a concise discussion of the term biomarker by Califf (90). Thus, rather of being a unique biomarker antibody for SLE, the antibodies demonstrate clinical associations with SLE, infections and malignancies, aside from appearing sporadic in other disorders (13).

In SLE, anti-dsDNA antibodies are pathogenic in context of lupus nephritis (11, 79), dermatitis (82, 91), and in certain forms of cerebral lupus (27, 83, 92). Whether these pathogenic pathways are determined by cross-reaction with non-DNA structures (see Table 1 for examples) or by homologous recognition of chromatin/dsDNA exposed in e.g., glomeruli (11, 93, 94), skin basement membranes (82), or in the brain (27) is still not firmly established. These clinical origins of anti-dsDNA antibodies has not been seriously considered in the classification criteria, nor in the relevant literature on anti-dsDNA antibodies and SLE [discussed in (1, 2, 5, 13, 14, 39)].

**Table 1:** Examples of anti-dsDNA antibodies that cross-react with non-DNA structures.

**Anti-dsDNA antibody cross-react with**	**References**
α-actinin	(17, 18)
Laminin	(19, 20)
C1q	(21)
Several cross-reactive activities presented at “Fifth International Workshop on anti-DNA anti-bodies in London 2002 to highlight relevant properties of pathogenic anti-DNA antibodies”	
	(22)
Phospholipids	(23)
Nucleosomes	(24)
Platelet integrin GPIIIa 49–66	(25)
TLR 4	(26)
NR2 glutamate receptor	(27)
Cell surface proteins^***^	(28)
Ribosomal P protein	(29)
Collagen IV	(30)
Pneumococcal antigen	(31)
EBNA	(32)
Entactin	(33)
Entactin^*^	(34)

* *Mono-specific anti-Entactin antibody is included to suggest a control non-cross-reactive antibody to determine if dsDNA as a cross-reactive specificity is required to gain pathogenic potential*.

### Anti-dsDNA Antibodies: Recognition of Disparate Unique dsDNA Structures and Not Simply dsDNA (a Review of Relevant Literature)

In the modern history of DNA discoveries, different forms of DNA structures have been described in highly focused research projects directed at describing what DNA is, which DNA structures exist, and their role in facilitating and regulating transcription of genes. Therefore, the second principal problem to be considered is that the antibodies are presented as if they constitute one specificity for dsDNA— “The anti-dsDNA antibody” (1, 2, 5). “The anti-dsDNA antibody” is not an unambiguous term, and the antibody reflects specificities to a variety of structures far beyond the canonical double helix structure. These structures represent the contexts in which dsDNA is presented to the immune system. The term “The anti-dsDNA antibody” comprises specificities toward ssDNA (95), Z DNA [left-handed dsDNA (96–99)], bent and elongated B DNA [right-handed dsDNA (100, 101)], diverse ss- and ds-RNA sequences and RNA-DNA double-strand hybrids (102, 103), folded and unfolded cruciform DNA structures (104, 105), bacterial DNA (106, 107), and finally different forms of viral dsDNA (108–110) that differ from mammalian dsDNA structurally and serologically (85, 110). Among these individual DNA structures, the most enigmatic in an auto-immunogenic context is the mammalian B form of dsDNA, since many of the other DNA structures were proven immunogenic, but this was not the case with mammalian B DNA. Therefore, over decades B DNA was considered non-immunogenic [(99, 111, 112), reviewed in (13)]. Anti-dsDNA antibodies further cross-react with a large panel of proteins and phospholipids. This heterogenous group of antigens targeted by anti-dsDNA antibodies are exemplified in Figure 2B and Table 1. The referred antibody specificities have been detected in natural situations (13), while antibodies have at least been raised experimentally to cruciform DNA structures (105).

One relevant question in this regard is whether fine molecular DNA antibody-specificities differ between their appearance in infections, malignancies and in SLE, as some antibodies may appear depending on the clinical situation, as is demonstrated for experimental induction of Z dsDNA but not B dsDNA specific antibodies in mice (99) although both appear in SLE. A similar observation relates to the fact that the frequency of antibodies to elongated mammalian dsDNA, as nucleosomal linker dsDNA, is higher than antibodies to bent dsDNA as in the core mononucleosome, both present on the same chromatin structure (101, 113, 114) or to bent dsDNA as in the plasmid of *Crithidia luciliae* (100).

## Anti-dsDNA Antibodies: Assay Conditions do Not *per se* Determine Levels of Antibody Avidities, but Reflect Disparate Unique dsDNA Specificities

The term “The anti-dsDNA antibody” has over decades shaped the concept that different assay systems detect antibodies possessing different avidities to dsDNA, but not different molecular or structural dsDNA specificities. This problem has not been considered when discussing binding of antibodies to dsDNA in principally different antibody assay systems. This conflict is demonstrated in Figure 2B. Antibodies binding the “ssDNA/dsDNA” structures are in the figure classified into 4 main categories with 5 subcategories for mammalian dsDNA, 2 for infectious dsDNA and 2 for cross-reaction of anti-dsDNA antibodies with renal and non-renal proteins and phospholipids (Figure 2B, see Table 1 for details on the latter categories). These categories and subcategories are examples of pertinent diversity of dsDNA structures recognized by this family of antibodies [see e.g., (115–117), all specific for functional DNA structures or infectious-derived chromatin/DNA].

Antibodies for these dsDNA structures have been identified by conventional assay systems, like ELISA-based detection of anti-dsDNA antibodies against dsDNA in physiological salt; in high salt (Z dsDNA); cruziform dsDNA; bacterial and viral dsDNA [summarized in (10, 13)]. The idiom that anti-dsDNA antibodies are presented in a singular form (“The dsDNA”) must be challenged by the comprehensive structural recognition diversity. This clearly opens for individual specificities generated by different functional/structural states of dsDNA rather than individual avidities (either low or high) linked to different assay conditions. For example, if an antibody binds dsDNA in 2M NaCl, it binds to a structure shaped in 2M NaCl; the Z dsDNA (99, 118–121), and not because it has a high avidity over-winning the strength of the high salt concentration as in the Farr assay (122–124). This is also relevant for binding of other proteins to dsDNA structures in various salt concentrations (125–127). This difference is also evident from the fact that it is easier to experimentally induce antibodies to Z dsDNA than to B dsDNA (99). Similarly, antibodies that bind elongated dsDNA as in ELISA and antibodies that bind bent dsDNA as in *Crithidia luciliae* or in the core nucleosome may possess the same level of avidities, but the antibodies recognize different structures, elongated vs. bent dsDNA (128).

Thus, interpretation of data in Figure 2B demonstrate that assay systems for anti-dsDNA antibodies detect specificities that may have no or high potential impact as biomarker for SLE. Their individual impact as classification criteria for SLE has, however, not been investigated. This problem needs to be solved in order to select the proper assays for clinically relevant anti-dsDNA antibodies.

### Anti-dsDNA Antibodies: Immunogenic Origin—Facts and Controversial Hypotheses

**Figure 3:**
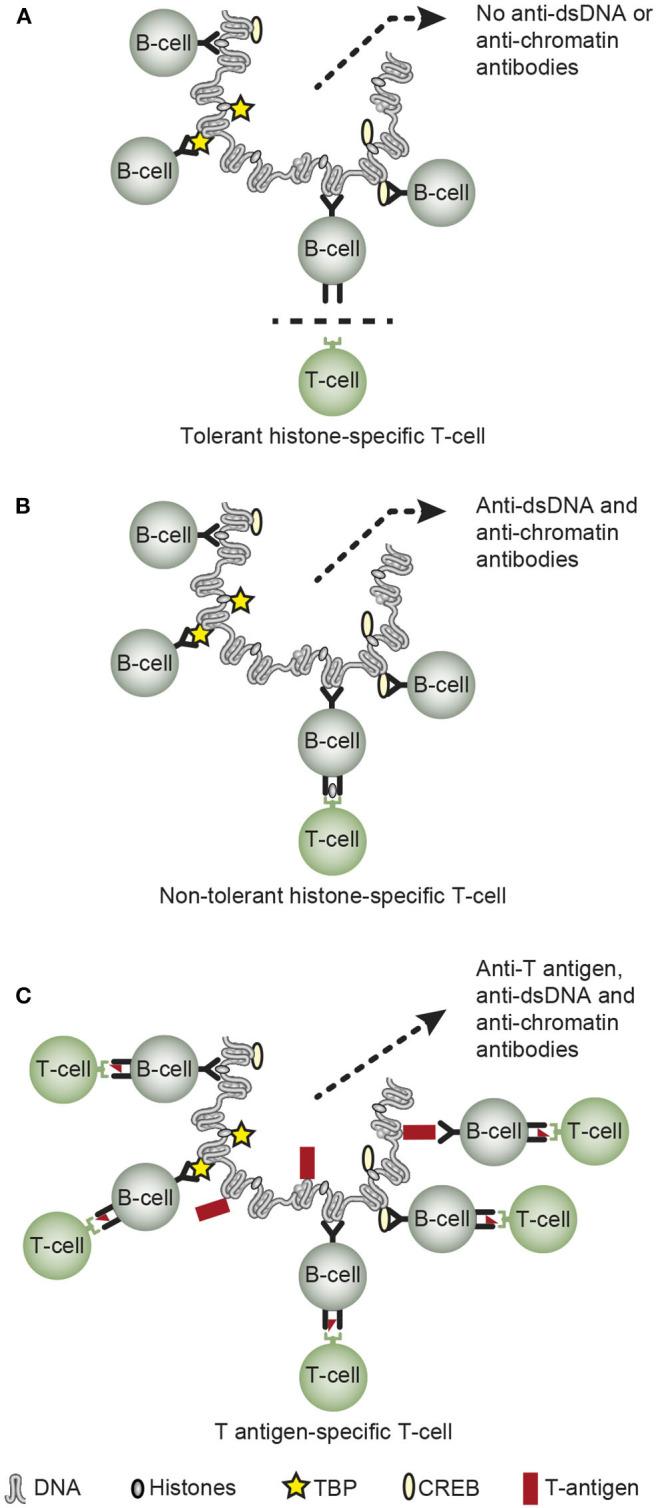
Principal basic problems attempting to describe how or why chromatin/dsDNA execute their immunogenic potential *in vivo*. Concerning the origin of anti-dsDNA antibodies, they are not induced on a normal immune background by exposure of pure autologous chromatin [**A**, (7, 111, 112, 129, 134, 136)], although autologous chromatin may gain immunogenic power in situations where helper T cell tolerance is truly terminated as demonstrated in SLE [**B**, (141, 144–146)]. Infections may provide chromatin-binding non-self-proteins that allow cognate interaction of chromatin/dsDNA-specific B cells and non-tolerant helper T cells specific for the infectious chromatin-associated protein [**C**, see Table 2 for examples (15, 49, 50, 86, 147)]. However, the possible immunogenicity of chromatin in malignancies has not been explored in depth, although infections as are predisposed for in cancers may encompass a model that is analogs to the one presented in **(C)** (**C** is reprinted with permission from Figure 5 in Rekvig (3)].

The third principal problem considered for the anti-dsDNA antibodies relates to its biological origin—what imposes production of these antibodies. Normally, mammalian dsDNA is non-immunogenic (111, 129, 130). Tolerance in a normal homeostatic situation is maintained at several biological check-point levels. B cells specific for dsDNA above a certain level of affinity are deleted in the bone marrow (131, 132); their antigen receptors are edited, with loss of affinity for dsDNA [(133–135), see a general discussion in (136)]; or they may appear anergic and non-functional (137, 138). Tolerance is also controlled by T helper cells. CD 4+ T cell deletion prevents autoimmunity, and CD 4+ T cells targeting chromatin-derived peptides are normally anergic (139). This is evident from experiments demonstrating that anergic CD 4+ T cells can be rendered functional in response to IL-2 (140, 141), thus helping B cells to transform into antibody-producing plasma cells. If such cells are deleted (142, 143), this will prevent B cells to receive competent CD4+ T cell signals to be transformed into antibody-producing plasma cells. This situation is presented in a simplified version in Figure 3A, where tolerant (anergic or deleted) T cells are indicated.

In SLE, however, autologous chromatin may gain immunogenic power because e.g., histone-specific CD 4+ T cell tolerance is truly terminated [Figure 3B, as described in (144–146, 148, 149)]. Termination of histone-specific CD4+ T cell tolerance is also easily achieved in experimental contexts (140, 141, 150), thus demonstrating that CD4+ T cell autoimmunity to chromatin-derived peptides is a latent property of the normal immune system (141).

Data have been demonstrated that infections may provide chromatin-binding proteins allowing cognate interaction of chromatin-specific B cells and non-tolerant helper T cell specific for the infectious chromatin-associated proteins [Figure 3C, see e.g., (13, 15, 49, 50, 86, 147)]. This model is denoted the hapten-carrier model for *in vivo*-induction of anti-chromatin/anti-dsDNA antibodies.

Anti-dsDNA antibodies can also be produced through a mechanism known as molecular mimicry (151–154), and the central and important study by Lafer et al. published in 1981 opened this topic for further studies of molecular mimicry as a potential driving force for appearance of anti-dsDNA antibodies (23).

Which of these models (described in Figures 3B,C) are operational in malignancies have not been investigated in depth. Since malignancies are complicated by viral and bacterial infections (155–157), this apprehension may hypothetically authorize the hapten-carrier model for infection-induced anti-dsDNA antibodies also in malignant diseases [as indicated in Figure 3C, exemplified by the role of e.g., the viral dsDNA-binding polyomavirus T antigen (50, 147)]. It is therefore tempting to assume that infections in malignant diseases may encompass a model in analogy to the one presented in Figure 3C—the hapten-carrier model, as explained in the next section.

### Anti-dsDNA Antibodies: *In vivo* Expression of Virus-Derived, DNA-Binding Proteins Render Chromatin Immunogenic— Evidence for the Hapten-Carrier Model

In Table 2, data are presented providing evidences that *in vivo* expression of single dsDNA/chromatin-binding viral proteins instigate the production of anti-dsDNA, anti-histone, and anti-transcription factor antibodies like anti-TATA-binding protein (TBP), anti-cAMP-response element binding protein (CREB) antibodies in accordance with the hapten (dsDNA) -carrier (viral dsDNA-binding protein) model. Molecular mimicry is less probable as explanation for production of anti-dsDNA in context of these experiments, since *in vivo*-expression of a mutated SV40 T antigen, the SLT155_T>S_, rendered the SV40 T antigen non-dsDNA-binding and did not elicit production of anti-dsDNA or anti-histone antibodies [Table 2, (147)]. Expression of this mutant protein resulted, however, in antibodies to T antigen (Table 2). Summarizing the models described in Figures 3A–C and Table 2, tolerance is maintained in a normal individual, terminated in SLE patients and lupus-prone mice, and tolerance is also terminated in context of certain (complicating) infections.

**Table 2:** Experimental expression of single viral DNA-binding proteins in context of plasmid injections, promote production of anti-dsDNA antibodies and to a variety of chromatin proteins.

**Plasmid**	**promoter**	**Expressed** **antigen**	**Anti-T antigen** **antibodies**	**Anti-dsDNA** **antibodies**	**Anti-Histone antibodies**	Anti-TBP^*^	Anti-CREB^**^	**References**
pRSV-BKT	RSV LTRt	Pyv T ag	681 ± 40 ^***^	715 ± 69	557 ± 112	318 ± 99	402 ± 91	(50, 147)
pRcCMV-BLT	HCMV ie-1	Pyv T ag	1282 ± 186	871 ± 53	763 ± 87	468 ± 254	552 ±186	(50, 147)
pBS-BLT	None	Not expressed	Not detected	Not detected	Not detected	Not detected	Not detected	(50, 147)
pRcCMV-SLT155	HCM ie-1	SV 40 T ag	573 ± 34	360 ± 46	259 ± 52	Not tested	Not tested	(50, 147)
pRcCMV-SLT155_T>S_	HCM-ie-1	SV 40 T ag Mutant non-dsDNA binding	442 ± 44	29% ± 2, 5^****^	Not detected	Not tested	Not tested	(50, 147)
pRA 17	SCM-ie 1	EBNA 1 (69.7 kDa)	Not tested	EBNA-1, dsDNA, Sm	Not tested	Not tested	Not tested	(49)

*All reported experiments are performed in BALB/c mice*.

* *TATA-binding protein*.

** *cAMP-response element binding protein*.

*** *The results are given as titers. These were determined by ELISA analyses of the induced serum antibodies aimed to quantify autoantibodies in mice injected by various T antigen expressing/non-expressing plasmids. The titers were determined from 2-fold dilution curves starting from dilution 1/100. The titers were defined as the reciprocal value of the dilution giving 50% of maximal binding to wells, as determined by individual reference sera (147)*.

**** *These sera gave only marginal binding values as their binding at 1/100 dilution gave only 29% of the binding of a reference serum included in these assays*.

### Anti-dsDNA Antibodies: Tolerance to Chromatin and the Role of Autologous Chromatin-HMGB1 Complex and of DNase 1L3 Gene Deficiency in Promoting Anti-dsDNA Antibody Responses

There is still, aside from generation of hypotheses (158–161), no consensus as to whether pure autologous chromatin is rendered immunogenic in context of reduced clearance of apoptotic chromatin from dead and dying cells. Furthermore, no firm evidence has been provided that exposure of neutrophil extracellular traps (NETs) induce antibodies to dsDNA [see e.g., an insightful and still relevant discussion by Pieterse and van der Vlag (162)]. These hypotheses have been discussed over the last 2 decades, although sound experimental biologically relevant evidences are still lacking [see a discussion in (3, 13, 161, 162)].

Two independent observations may, however support the view that autologous chromatin possesses auto-immunogenic potential. Urbonaviciute et al. demonstrated that anti-dsDNA/chromatin antibodies are induced upon exposure of the high mobility group box protein 1 (HMGB1) tightly attached to chromatin in apoptotic cells (150). In another study, a null mutation in the DNase1L3 gene was described in SLE patients with lupus nephritis (163). This deficiency correlated with production of anti-dsDNA antibodies and lupus nephritis. In agreement with the observational study in familiar SLE, mice with experimentally deficient expression of the DNase 1L3 gene developed analogous anti-dsDNA antibodies and lupus nephritis (164). Thus, in DNase 1L3 gene deficient individuals extracellular degradation of chromatin is reduced and this deficiency correlates with promotion of anti-dsDNA antibodies. Thus, clearance deficiency of chromatin due to DNase 1L3 deficiency or release of extracellular chromatin in complex with HMBG1 from apoptotic cells, are two potential sources of complex autologous immunogens in both mice and humans.

## Anti-dsDNA Antibodies and Lupus Nephritis

In the next section new aspects and interpretive problems will be discussed in attempts to understand the link between anti-dsDNA antibodies, lupus nephritis and SLE. This is on one hand easy to do when considering the enormous amount of studies accepting this linkage, but on the other hand difficult if basic scientific data are considered critically.

### SLE and “The Anti-dsDNA Antibody” —Clinical and Biological Contexts

Anti-dsDNA antibodies were first described in an infectious context in 1938–1939 (165–167), while in an autoimmune context in 1957 (168–171). Despite considerable scientific efforts we have not reached consensus on four fundamental aspects of anti-dsDNA antibodies in SLE. These aspects are comprised by four dogmatic areas: Their (i). Origin, (ii). Structural DNA specificities, (iii). Pathogenic impact, and *iv*. Assumed link to SLE [see discussions above and in (3, 11, 13, 14, 172)].

### Anti-dsDNA Antibodies—Specificity Critically Determines Nephrogenicity and May Also Affect Alveolitis and Dermatitis?

**Figure 4:**
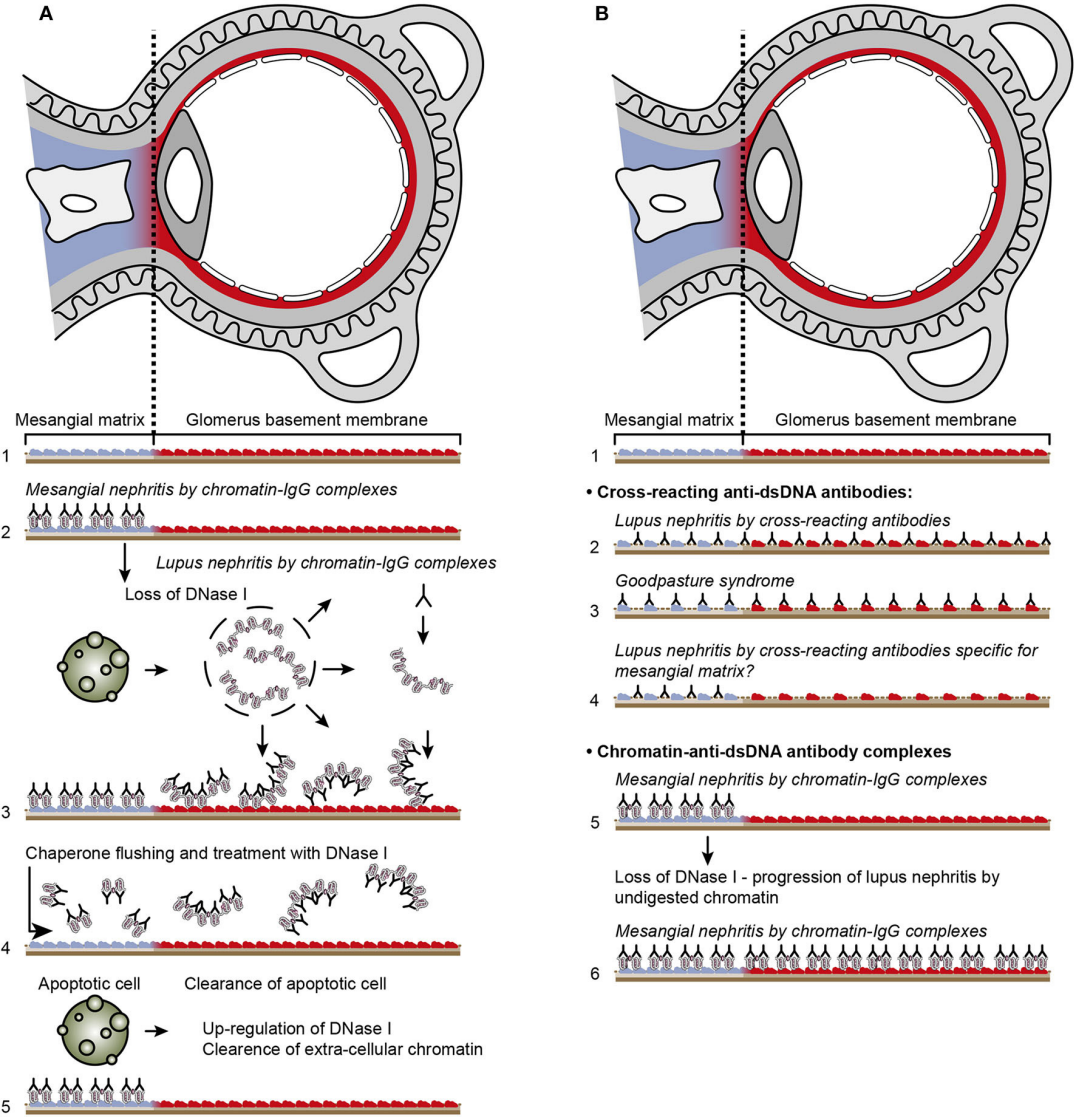
Principal problems to be solved before the chromatin or the cross-reactive model for lupus nephritis can be settled. In **(A)**, the chromatin model is presented. On top, a principal presentation of the architecture of a glomerulus is described. In line 1, the mesangial matrix (blue) and its transition into the GBM (red) is principally demonstrated. In a classical progression of lupus nephritis (177, 178), chromatin-IgG complexes deposit in the mesangial matrix and form the early mesangial nephritis (line 2). One consequence of this limited inflammation is silencing of the renal endonuclease DNase 1, a consequent reduced fragmentation of chromatin from dead cells, and a subsequent deposition of large chromatin fragments in complex with IgG within the GBM [line 3, (178, 179)]. This forms the process that promote glomerular inflammation and progression of lupus nephritis into end stage disease [discussed in (11)]. Silencing of DNase 1 expression in this situation is unique to the kidney and does not occur in other organs (179). Since chromatin-IgG complexes bind laminins and collagens in the GBM with relatively high affinity (180), and are released locally in the glomerulus, these observations may explain the canonical progression of lupus nephritis as described by Weening et al. (177). This process may have therapeutic consequences, since chromatin prone to be deposited in GBM may be removed by flushing kidneys with the negatively charged heparin or other analogous chaperone molecules [line 4, (181)^5^], and theoretically, the process may be interrupted upon upregulation of renal DNase 1 expression [line 5, (181)]. In **(B)**, the glomerulus architecture is organized as in **(A)**, and the matrix-GBM transition is principally illustrated (line 1). In the cross-reacting model, cross-reacting anti-dsDNA antibodies bind intrinsic glomerular structures like entactin, laminin or collagen (line 2, see also data in Table 2). Since these antibodies may bind ligands shared by mesangial matrix and GBM, the antibodies are expected to bind simultaneously in the mesangial matrix and in the GBM (line 2). Therefore, the cross-reactive antibodies might well-initiate a glomerular inflammation more similar to the renal inflammation in Goodpasture syndrome (line 3) than to the stepwise progression of lupus nephritis as illustrated in **(A)**, lines 2 and 3. This consequence has not been considered in the relevant literature. One possible exception for this Goodpasture-like inflammation would be an early production of antibodies specific for a ligand unique for the matrix (suggested in line 4) and that the mesangial nephritis promoted by this particular antibody incites an inflammation that down-regulates renal DNase 1 and a subsequent exposure of undigested chromatin fragments in complex with IgG anti-chromatin antibodies in GBM. This would promote the evolution of progressive lupus nephritis. In contrast to this hypothetical model, lines 5 and 6 summarize progressive lupus nephritis according to the chromatin model. These principally conflicting models are summarized in **(A)**, lines 2 and 3 for the chromatin model, and in **(B)**, line 2 for the cross-reactive model [This figure is a revised and extended version of Figure 4 in Rekvig et al. (182) with permission from Elsevier (License number 4832930988362)].

**Figure 5:**
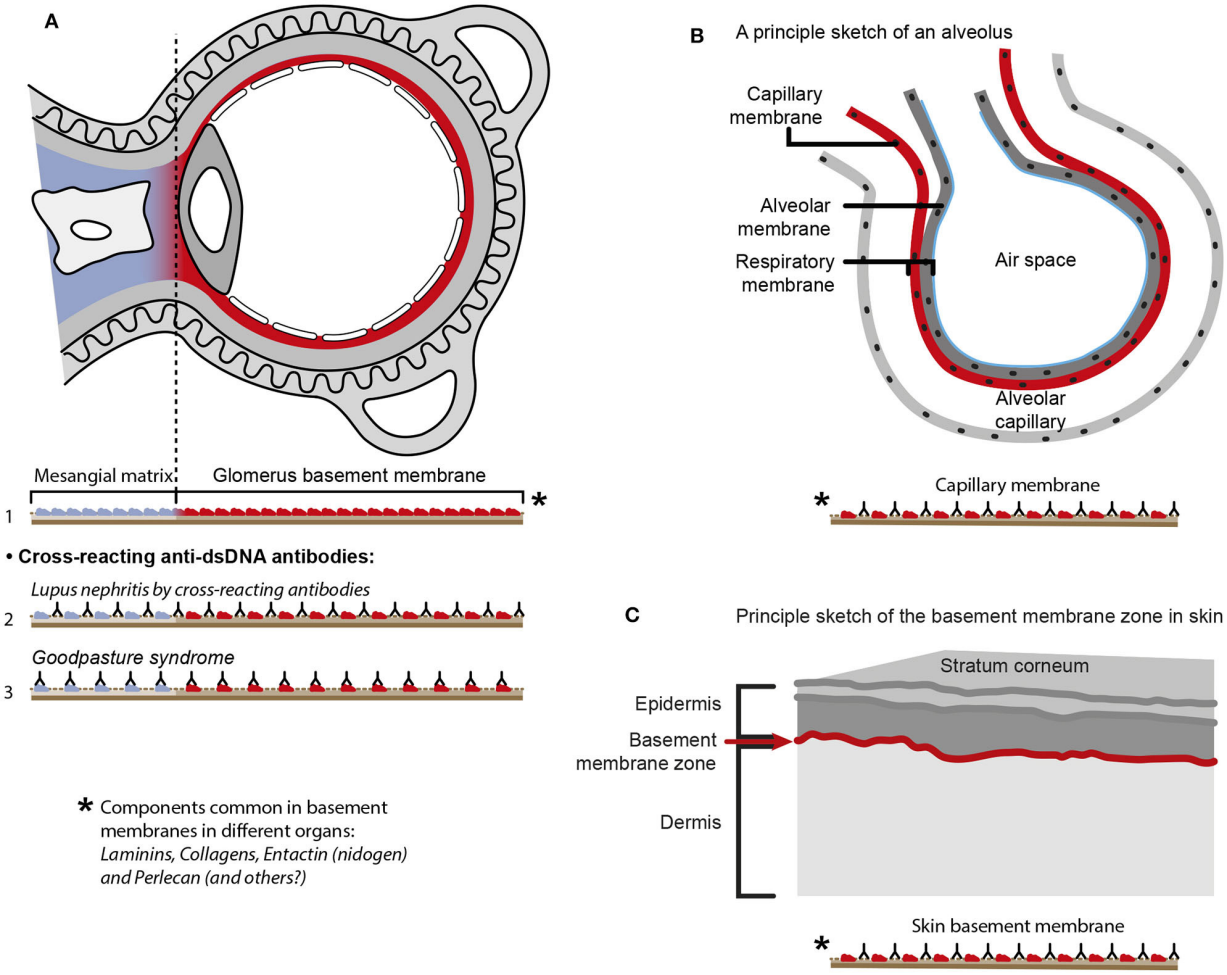
Principal problems linked to the cross-reactive model for lupus nephritis. The cross-reactive model inherits another provoking problem. Laminins, entactin and collagens are obligate constituents in all basement membranes. This is relevant for basement membranes in glomeruli [see (11) for discussion], alveoli (187) and skin (188). Accordingly, one should expect affection of glomeruli **(A)**, alveoli **(B)** and skin **(C)** in analogy to Goodpasture syndrome [glomeruli and alveoli (189, 190)] and autoimmune skin diseases (191–193). Surprisingly, in context of studies on the impact of cross-reactive anti-dsDNA antibodies as a model for pathogenesis of lupus nephritis, the involvement in other organs has not been discussed or considered in the relevant studies. Observational and experimental studies argue against this theoretical model. Analyses of nephritic glomeruli by electron microscopy (EM), immune EM (IEM), co-localization IEM, TUNEL co-localization IEM, allowed clear indications that *in vivo* bound IgG were observed in electron dense structures (EDS) localized in the matrix and GBM [(194–196) summarized and discussed in (11)]. These EDS were TUNEL positive, and bound antibodies against histones, transcription factors and dsDNA added to sections *in vitro*. Because of the problematization of how “The anti-dsDNA antibody” promotes lupus nephritis, questions that are illustrated in Figure 4, should be considered and focused on in future studies on the distinct autoimmune pathogenesis of nephritis [**A** in this figure is a truncated reprint of Figure 4 in Rekvig et al. (182) with permission from Elsevier (License number 4832930988362)].

Consensus has been established that anti-dsDNA antibodies promote lupus nephritis. How they do so are still controversial. The schisms divide scientists into two main interpretive groups. One assumes that the antibodies bind chromatin exposed in the kidneys (11, 173–176). This model is presented in Figure 4A. The other mainstream model implies that nephritogenic anti-dsDNA antibodies cross-react with intrinsic glomerulus basement membrane (GBM) constituents (illustrated in Figures 4B, **5**). Which of the many autoantibodies described in SLE (183) involved in promotion of lupus nephritis remain, however, elusive. The cross-reacting antibodies assumed to be implicated in lupus nephritis recognize among many ligands laminin (19, 184, 185), α-actinin (17, 18, 186), C1q (21), and entactin [(30, 33), Table 1].

There is, however, one central problem in these studies. Each of them focuses on one cross-reactive pattern and conclude that the actual cross-reaction correlates with lupus nephritis. Surprisingly, no discussion is presented that require a mutual multicenter study that compare the different cross-reactions in one cohort of lupus nephritis patients. This approach is awaited because nephritogenic-prone cross-reactions can be identified, and those that do not correlate with nephritis can be separated as unrelated epiphenomena. It would be important also to test if mono-specific antibodies not recognizing dsDNA, like those monospecific for e.g., laminin, or α-actinin, have the potential to promote lupus-like nephritis, as is suggested for the mono-specific anti-entactin antibody presented in Table 2.

To understand the basis for these problems, it is necessary to understand the unique processes of the two dominating models for lupus nephritis. In Figure 4A, the chromatin model is presented. On top, a principal presentation of the architecture of a glomerulus is illustrated and in Line 1 a principal transition of the mesangial matrix into the GBM is indicated.

In a classical progression of lupus nephritis, as described in (NZBxNZW)F1 mice (177, 178) chromatin-IgG complexes deposit in the mesangial matrix and form the early mesangial nephritis (Figure 4A, line 2). One consequence of this limited inflammation is silencing of renal DNase 1, reduced fragmentation of chromatin from dead cells, and subsequent accumulation of large chromatin fragments in complex with IgG in the GBM [line 3, (178, 179)]. This process forms the basis for a systemic glomerular inflammation and progression of lupus nephritis into end stage renal disease. Silencing of DNase 1 expression in this situation is unique to the kidney and does not occur in other organs (179). Notably, the mesangial matrix and the GBM share constituents like laminins, collagens and entactin. As chromatin-IgG complexes bind laminins and collagens with relatively high affinity (180), and are released locally in the glomerulus, these observations may explain the canonical progression of lupus nephritis from mesangial nephritis into end-stage disease as described by Weening et al. (177). This process may have specific therapeutic consequences, since chromatin prone to be deposited in GBM may be removed by flushing kidneys with heparin or other analogous chaperone molecules [Figure 4A Line 4, (181)], and theoretically, the process will assumedly be interrupted upon upregulation of renal expression of DNase 1 [(181), as indicated in Figure 4A, line 5]. Such experimental approaches are awaited.

In Figure 4B, the architecture of the glomerulus is repeated, and the transition of the mesangial matrix into the GBM is indicated (Figure 4B, line 1). Cross-reacting anti-dsDNA antibodies bind non-dsDNA, intrinsic mesangial matrix and GBM structures like entactin, laminin or collagen (Figure 4B, line 2); thus, these antibodies may from theoretical arguments simultaneously bind ligands shared by the mesangial matrix and GBM. If this model is correct, the cross-reactive antibodies might well initiate a glomerular inflammation similar to the renal inflammation in Goodpasture syndrome (Figure 4B, Line 3). The model may indicate that mesangial nephritis does not precede progressive lupus nephritis, but appear simultaneously. This consequence of a cross-reaction has not been considered in the literature.

One possible exception for this would be an early production of antibodies specific for a ligand unique for the matrix (suggested in Figure 4B, line 4) or that the mesangial nephritis promoted by this particular antibody incites an inflammation that down-regulates renal DNase 1 and subsequent exposure of large chromatin fragments in GBM and thereby the evolution of progressive lupus nephritis. In contrast to this hypothetical model, Figure 4B lines 5 and 6 summarize progressive lupus nephritis according to the chromatin model. These principally conflicting models are summarized in Figure 4A, lines 2–3 for the chromatin model, and Figure 4B, line 2 for the cross-reacting model.

The cross-reactive model also inherits another provoking problem that is not regarded in the literature. Since e.g., laminins, entactin, collagens, and other ligands are obligate constituents in all basement membranes, this is relevant also for basement membranes in glomeruli [discussed in (11)], alveoli (187) and skin (188). Accordingly, one would expect affection of glomeruli (Figure 5A), alveoli (Figure 5B) and also skin (Figure 5C) in analogy to Goodpasture syndrome [glomerulonephritis and alveolitis (189, 190)], and to autoimmune skin diseases (191–193, 197). Surprisingly, in context of studies on the impact of cross-reactive anti-dsDNA antibodies as central in the pathogenesis of lupus nephritis, the involvement in other organs has not been considered in relevant studies.

### Concluding Remarks and Four Concise Hypotheses

SLE is a complex serious disease considered to rely on an autoimmune pathogenesis. One central question arises from the discussions above: Is SLE with nephritis another syndrome than SLE without nephritis? And are the same clusters of classification criteria, and the same sets of biomarkers linked to SLE with and without nephritis or with or without anti-dsDNA antibodies informing about the same fundamentals of the disease? We do not need more classification criteria in the aftermath of those tentatively identified till now. For now, we first have to develop an understanding why they appear in clusters and thereby why they define the syndrome SLE. Today, these problems are hidden from our perspective on SLE in our search for new classification systems for SLE. We need a penetrating and better theoretical model for SLE, to generate a basis for new and stringent cohort studies. The discussions given above on classification systems for SLE, anti-dsDNA antibodies and phenotypes of lupus nephritis must be imperative to develop new concise and testable hypotheses.

The following hypotheses may be considered.

- Analyzing cohorts of SLE patients selected by ACR or SLICC criteria will identify a larger spectrum of deviating clinical and biological parameters than homogenous cohorts of patients selected based on e.g., proteinuria and anti-dsDNA antibodies.
- The “Anti-dsDNA antibody” has lower impact as a classification criterium than anti-dsDNA antibodies specific for certain unique dsDNA structures.
- Different assay systems detect antibodies with different structural dsDNA specificities and not different avidities of the antibodies detected in the individual assay systems, thus this may result in different phenotypic presentations of SLE.
- If crossreacting anti-dsDNA antibodies bind renal basement membrane structures like laminin, entactin and collagen, the probability that they bind basement membranes in other organs is high. If not, one may question whether cross-reaction is of clinical significance.

In sum, SLE remains an enigmatic disease despite (or because) implementing new classification criteria; anti-dsDNA antibodies in clinical medicine are still poorly defined; lupus nephritis pathogenesis needs to be defined with respect to specificity of nephritogenic anti-dsDNA antibodies: Specificity for dsDNA or cross-reacting renal antigens.

## Data Availability Statement

The present article is a review on hypotheses and theories related to murine and human SLE and lupus nephritis. All data are taken from original published studies approved by relevant ethical committees.

## Ethics Statement

The present manuscript is a review on hypotheses and theories related to murine and human SLE and lupus nephritis. All data are taken from original published studies approved by relevant ethical committees. The patients/participants provided their written informed consent to participate in this study.

## Author Contributions

The author confirms being the sole contributor of this work and has approved it for publication.

## Conflict of Interest

The author declares that the research was conducted in the absence of any commercial or financial relationships that could be construed as a potential conflict of interest.

## Footnotes

^1^Triangular in this context: Relating to, or involving three elements (Merriam-Webster).

^2^“Koch”. Random House Webster's Unabridged Dictionary.

^3^Positivism: A philosophical system founded by Auguste Comte, concerned with positive facts and phenomena, and excluding speculation upon ultimate causes or origins.

^4^Hermeneutics is the theory and methodology of interpretation, especially the interpretation of biblical texts, wisdom literature, and philosophical texts.

^5^Jenny Buckland. Therapeutic targeting of chromatin in lupus nephritis? Research Highlights *Nat Rev Rheum*. (2011) 7:132.
